# PP1A-Mediated Dephosphorylation Positively Regulates YAP2 Activity

**DOI:** 10.1371/journal.pone.0024288

**Published:** 2011-09-01

**Authors:** Pei Wang, Yujie Bai, Bangrong Song, Yadong Wang, Dong Liu, Yongqiang Lai, Xiaolin Bi, Zengqiang Yuan

**Affiliations:** 1 State Key Laboratory of Brain and Cognitive Sciences, Institute of Biophysics, Chinese Academy of Sciences, Beijing, China; 2 College of Life Sciences, Graduate School of the Chinese Academy of Sciences, Beijing, China; 3 Department of Cardiac Surgery, Anzhen Hospital at Capital Medical University, Beijing, China; 4 Laboratory for Biological Effects of Nanomaterials and Nanosafety, Institute of High Energy Physics, Chinese Academy of Sciences, Beijing, China; Erlangen University, Germany

## Abstract

**Background:**

The Hippo/MST1 signaling pathway plays an important role in the regulation of cell proliferation and apoptosis. As a major downstream target of the Hippo/MST1 pathway, YAP2 (Yes-associated protein 2) functions as a transcriptional cofactor that has been implicated in many biological processes, including organ size control and cancer development. MST1/Lats kinase inhibits YAP2's nuclear accumulation and transcriptional activity through inducing the phosphorylation at serine 127 and the sequential association with 14-3-3 proteins. However, the dephosphorylation of YAP2 is not fully appreciated.

**Methodology/Principal Findings:**

In the present study, we demonstrate that PP1A (catalytic subunit of protein phosphatase-1) interacts with and dephosphorylates YAP2 *in vitro* and *in vivo*, and PP1A-mediated dephosphorylation induces the nuclear accumulation and transcriptional activation of YAP2. Inhibition of PP1 by okadiac acid (OA) increases the phosphorylation at serine 127 and cytoplasmic translocation of YAP2 proteins, thereby mitigating its transcription activity. PP1A expression enhances YAP2's pro-survival capability and YAP2 knockdown sensitizes ovarian cancer cells to cisplatin treatment.

**Conclusions/Significance:**

Our findings define a novel molecular mechanism that YAP2 is positively regulated by PP1-mediated dephosphorylation in the cell survival.

## Introduction

Hippo/MST signaling pathway has been delineated as a tumor suppressor pathway, which has been implicated in the diverse biological processes including cell proliferation, apoptosis, organ size control, and cancer development in both *Drosophila* and mammals [1], [2], [3]. MST1 and MST2 (mammalian STE-20 like kinase 1 and 2) are key components of the Hippo/MST1 tumor suppressor pathway [4], [5], and we have reported that MST1 mediates oxidative stress-induced neuronal cell death through phosphorylating FOXO3a at serine 207 [6]. Others and we also showed that PI3K/Akt and JNK regulate MST1 activation through protein interaction and phosphorylation during stress-induced cell death [7], [8], [9].

YAP2 was initially identified as a Src/Yes kinase associated protein [10]. Structurally, YAP2 contains one TEAD (TEF domain) binding domain in the N-terminus followed by two WW domains and PDZ binding motif in the C-tail [11], [12]. Multiple lines of evidences have shown that YAP2 functions as a transcriptional co-activator and downstream target of the Hippo/MST pathway in the biological processes of cell size control, proliferation and cell death[1], [2], [3], [13]. It has recently been shown that Mst1/2 ablation leads to hepatocellular carcinomas and the inhibition of YAP2 by MST kinases are an important mechanism in tumor suppression, especially in the progression of HCC (hepatocellular cancer) [14], [15], [16]. Interestingly, YAP2 was observed in nucleus in a panel of solid tumors, including liver, breast, ovary and prostate carcinomas [17], [18], [19], [20]. Together, these findings suggest that YAP2 functions as pro-survival factor and plays an important role in tumorigenesis. There is the striking conservation of Hippo pathway between *Drosophila* and mammals. Yki (Yorkie), the *Drosophila* orthologue of mammalian YAP2, promotes cell growth through transcriptionally co-activating Sd (Scalloped, *Drosophila* orthologue of mammalian TEAD) [1], [21], [22], [23], [24].

It has long been demonstrated that the post-transcriptional modifications (PTMs) of proteins are important for the regulation of its function, especially for the transcriptional factors and co-factors. The phosphorylation and ubiquitination of YAP2 has been reported [20], [25]. Recent studies showed that Lats phosphorylates YAP2 on serine 127 leading to its binding with 14-3-3 proteins [26], which results in cytoplasmic retention and transcriptional inhibition of YAP2. Consistently, the S127A (serine replaced with alanine) mutation retains YAP2 in the nucleus and increases its transactivation, resulting in the cell overgrowth and the epithelial mesenchymal transition [27], [28]. Taken together, the phosphorylation and dephosphorylation of YAP2 dynamically regulates its biological function. However, the dephosphorylation of YAP2 remains unclear.

The phosphoprotein phosphatase superfamily members include PP1, PP2A (PP2), PP2B (PP3), and PP4–7, among which PP1 is a major eukaryotic Ser/Thr protein phosphatase that involved in a variety of biological processes [29], [30]. TAZ, a mammalian YAP2 homologue [31], has been shown to be dephosphorylated by PP1A (catalytic subunit of PP1). By cooperating with ASPP2 (apoptosis stimulating protein of p53-2), PP1A increases TAZ-mediated gene expression [32], [33]. Most recently it has been reported that α-catenin regulates Yap1 activity and phosphorylation by modulating its interaction with 14-3-3 proteins and PP2A phosphatase [34].

In this study, we identify PP1A, as a component of YAP2 protein complex, regulates YAP2 function in the DNA damage-induced cell death. We found that PP1A and YAP2 interact with each other *in vitro* and *in vivo* and that PP1A dephosphorylates YAP2 at serine 127 and dissociates it from 14-3-3 binding, thus leading to its nuclear retention and transcriptional activation. We also observed that YAP2 expression inhibits ovarian cancer cell death induced by cisplatin treatment and the inhibition of PP1A with okadiac acid increases the phosphorylation and cytoplasmic translocation as well as the drug resistance capability of YAP2. Our findings demonstrate that YAP2 is functionally regulated by PP1A-mediated dephosphorylation.

## Results and Discussion

### PP1A Interacts with YAP2 *in vitro* and *in vivo*

**Figure 1:**
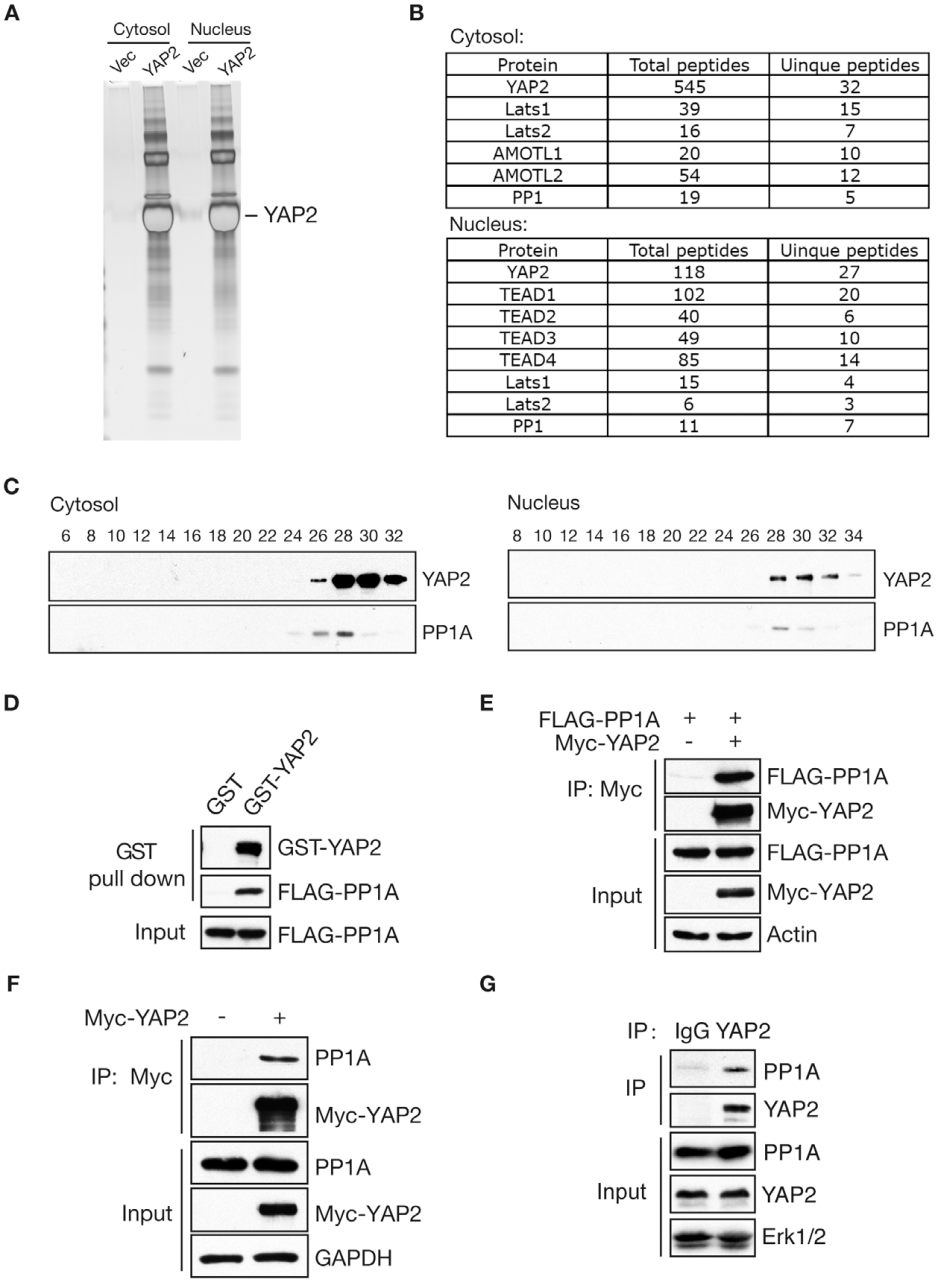
PP1A interacts with YAP2. A. Tandem affinity purification of YAP2 complex was performed as described in methods. The final HA-eluates from cytoplasmic and nuclear fractions were resolved on SDS-PAGE gels and silver-stained. B. PP1A was identified from the mass spectrometry analyses of both cytoplasmic and nuclear HA-eluates. The number of peptide hits for PP1A and several known YAP-interacting proteins are shown. C. Cytoplasmic and nuclear eluates were separated by 10%–40% glycerol gradient gels followed by Western blotting with anti-PP1 antibody. D. GST-pull down was carried out by incubating the recombinant GST-YAP2 or GST alone proteins with lysates of cells transfected with FLAG-PP1A and followed by immunoblotting with anti-FLAG antibody. E. Myc-immunoprecipitates from HeLa cells transfected Myc-YAP2 together with FLAG-PP1A were immunoblotted with anti-FLAG antibody. 2% input was blotted with anti- FLAG, Myc or actin. F. Myc-YAP2 plasmid or the control vector was transfected in HeLa cells and cell lysates were immunoprecipitated with anti-Myc antibody followed by immunoblotting with anti-PP1 antibody. 2% input was blotted with anti- PP1, Myc or GAPDH antibody, respectively. G. Endogenous interaction between YAP2 and PP1A.

To gain the further understanding of molecular mechanism underlying YAP2 regulation, we undertook the biochemical purification of YAP2. We generated stable HeLa cells expressing human YAP2 tagged with both the FLAG and haemagglutinin (HA) epitopes at its amino terminus. Cytoplasmic and nuclear extracts from these cells were subjected to sequential purification with anti-FLAG and anti-HA antibody columns. As shown in Figure 1A, several polypeptides were found to be specifically associated with the YAP2 (compared with the vector control), whose identities were determined by mass spectrometry. By mass spectrometry analysis, we identified several known YAP2-interacting proteins including Lats1/2, AMOTL1/2, ASPP1/2 and TEAD1-4 [35], [36], [37] in the cytoplasmic or nuclear fraction (Figure 1B). Notably, PP1A, an unappreciated YAP2 interacting protein, existed in both cytoplasmic and nuclear fractions (Figure 1B).

To further define PP1A as a YAP2 complex protein, we performed glycerol-gradient sedimentation and gel-filtration experiments. PP1A sedimented together with YAP2 (Figure 1C, peak fractions 26-30). We defined the interaction between PP1A and YAP2 in GST pull-down assays by using recombinant GST fusion proteins (Figure 1D). Upon expression in 293T cells, tagged YAP2 associated with either exogenous or endogenous PP1A (Figure 1E and 1F). Consistently, endogenous PP1A and YAP2 form a physical complex (Figure 1G). Taken together, these results suggest that YAP2 interacts with PP1A.

### YAP2 is Dephosphorylated by PP1A *in vitro* and *in vivo*

**Figure 2:**
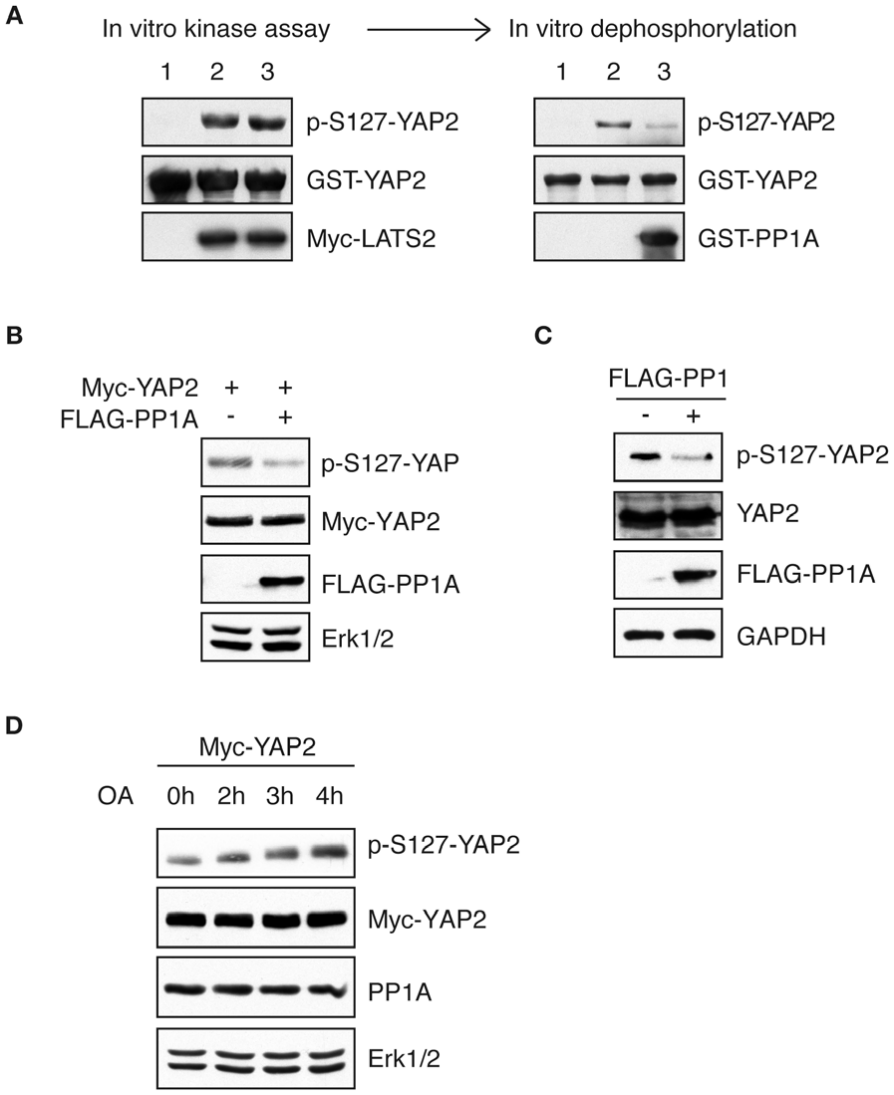
YAP2 is dephosphorylated by PP1A *in vitro* and *in vivo.* A. In vitro kinase assay and dephosphorylation assay was performed as described in methods. The recombinant GST-YAP2 proteins were incubated with immunoprecipitated LATS2 in the presence of cold ATP at 30°C for 30 minutes. The reaction products were analyzed by immunoblotting with anti-p-S127-YAP2 antibody. The kinase reaction mixture was incubated with the recombinant GST-PP1A at 37°C for 1 hour and followed by Western blotting with anti-p-S127-YAP2 antibody. PP1 dephosphorylates YAP2 at S127 *in vitro*. B. Lysates of HeLa cells transfected with Myc-YAP2 together with FLAG-PP1A or the control vector were immunoblotted with the indicated antibodies. C. Lysates of cells transfected with FLAG-PP1A or the control vector were blotted with anti-p-S127-YAP2 antibody. PP1A dephosphorylates endogenous YAP2 at serine 127. D. HeLa cells were transfected with plasmid encoding Myc-YAP2, and then treated with PP1A inhibitor OA for indicated time periods. The serine 127 phosphorylation levels of YAP2 were determined by Western blotting. E. HeLa cells were treated with OA for four hours and the cell lysates were analyzed with anti-YAP2 antibody.

Protein kinase Lats phosphorylates YAP2 at serine 127 and increases its association with 14-3-3 proteins, which leads to the cytoplasmic translocation and transcriptional inhibition of YAP2 [3], [38]. Due to the fact that phosphatase PP1A was identified as a YAP2 interacting protein, we asked whether YAP2 could be dephosphorylated by PP1. To address this issue, we performed the *in vitro* kinase assay by incubating the recombinant YAP2 proteins with the immunoprecipitated Lats2 kinase, followed by *in vitro* dephosphorylation assay using recombinant GST fusion protein encoding PP1A. We found that Lats2 phosphorylated YAP2 at serine 127 and PP1A dephosphorylated it (Figure 2A). We next expressed the exogenous PP1A together with YAP2 and PP1A expression reduced the pS127 levels of YAP2 in cells (Figure 2B). Expression of PP1A significantly reduced the endogenous phospho-S127 levels of YAP2 (Figure 2C). Furthermore, okadaic acid (phosphatase inhibitor) treatment increased YAP2 phosphorylation at serine 127 (Figure 2D). Taken together, these results support the conclusion that PP1A dephosphorylates YAP2 at serine 127.

### PP1A-mediated Dephosphorylation Promotes YAP2 Nuclear Accumulation

**Figure 3:**
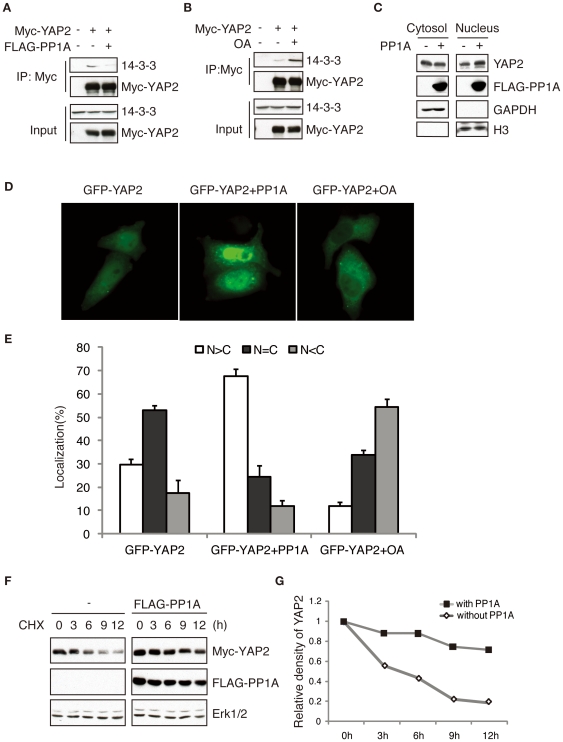
PP1A-mediated dephosphorylation promotes YAP2 nuclear accumulation. A. Myc-immunoprecipitates of HeLa cells transfected with Myc-YAP2 together with PP1A or control vector were immunoblotted with anti-14-3-3β antibody. PP1A expression inhibits the interaction between YAP2 and 14-3-3. B. Myc-immunoprecipitates of HeLa cells transfected with Myc-YAP2 plasmid or control vector and treated with or without OA were blotted with anti-14-3-3β antibody. Phosphatase inhibition increases the interaction between YAP2 and 14-3-3 proteins. C. Nuclear and cytoplasmic fractionation was performed from HeLa cells transfected with PP1A or control vector. The fractionated lysates were immunoblotted with anti-YAP2, GAPDH or Histone H3 antibody. PP1A increases the nuclear levels of YAP2. D. HeLa cells were transfected with the GFP-YAP2 and treated with or without 100 nM OA for 4 hours followed by fluorescent microscopy analysis. E. PP1A increases the nuclear accumulation of YAP2 and OA treatment induced YAP2 cytoplasmic translocation (*t*-test; n = 3, p<0.01). Data are represented as mean ± SEM. F. HeLa cells were transfected with Myc-tagged YAP2 together with FLAG-tagged PP1A expression plasmid or the control vector. 24 hours after transfection, cells were treated with 50 µg/ml Cycloheximide (CHX) for different time periods. Equal amounts of total protein lysates were subjected to immunoblotting. G. PP1A expression stabilized the protein level of YAP2.

It has been reported that serine 127 phosphorylation of YAP2 leads to the 14-3-3 binding and cytoplasmic translocation [3], [38]. It has been shown that 14-3-3 binding plays an important role in the regulation of YAP2 subcellular localization [38]. We examined the role of the PP1-mediated dephosphorylation of YAP2 in the inhibition of YAP2's interaction with 14-3-3 proteins. While expression of PP1A robustly disrupted the interaction of 14-3-3 proteins with YAP2 (Figure 3A), okadaic acid (OA) treatment increased the interaction between YAP and 14-3-3 (Figure 3B). To determine whether PP1A affect YAP2 subcellular localization, we performed the nuclear and cytoplasmic fractionation assays. As expected, PP1A expression increased the nuclear abundance of YAP2 proteins (Figure 3C). Consistently, PP1A expression reduced YAP2's nuclear accumulation and OA treatment increased the cytoplasmic translocation of YAP2 proteins (Figures 3D and 3E). Interestingly, we observed that the YAP2 protein level was increased when co-expressed with PP1A after protein synthesis inhibition by treating cells with Cycloheximide (CHX) (Figures 3F and 3G), which suggests that dephosphorylatioin of the YAP2 protein increases its stability. These results suggest that PP1A-mediated dephosphorylation of YAP2 triggers the dissociation of YAP2 from 14-3-3 proteins and leads to its nuclear accumulation.

### PP1A-induced Dephosphorylation of YAP2 Positively Regulates the Transcriptional Activity and Prosurvival Capability of YAP2

**Figure 4:**
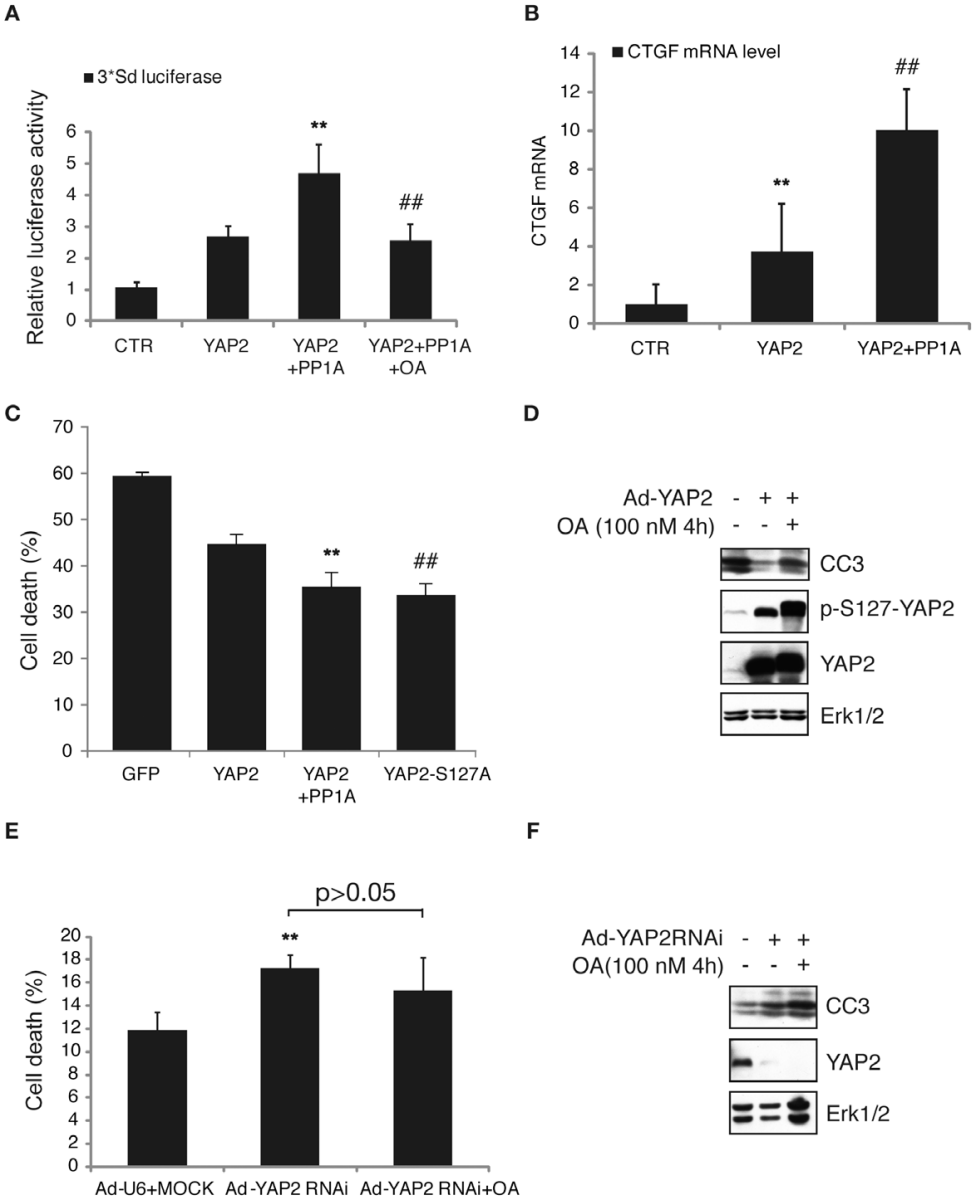
YAP2 dephosphorylation by PP1A regulates YAP2-mediated transcriptional activation and cell survival in ovarian cancer cell line. A. HeLa cells were transfected with the expression plasmid encoding YAP2 or PP1A plus the 3*Sd-luc luciferase reporter and renilla plasmids. The cell lysates were subjected to dual luciferase assay (*t*-test; n = 3, p<0.01). OA indicates that HeLa cells were treated with 100 nM OA for 4 hours. PP1A acts synergistically to activate YAP2-mediated transactivation (*t*-test; n = 3, p<0.01). B. Total RNA was isolated from HeLa cells transfected with the plasmids as indicated. The expression of CTGF was determined by relative quantitative RT-PCR and normalized to GAPDH. PP1A increases the expression of *CTGF* (*t*-test; n = 3, p<0.01). C. A2780 cells were transfected with YAP2 or YAP2-S127A plasmid together with or without PP1A. Cell death was analyzed after 24-hour cisplatin treatment followed by flowcytometry (*t*-test; n = 3, p<0.05). PP1A acts synergistically with YAP2 to enhance ovarian cancer cell survival. D. Lysates of cells infected with adenovirus YAP2 or the control vector treated cisplatin together with or without OA were immunoblotted with the antibody for cleaved caspase 3, p-S127-YAP2, YAP2 or Erk1/2, respectively. E. A2780 cells were infected with the adenovirus encoding YAP2 shRNA or control vector. Cell death was analyzed by flowcytometry after cisplatin treatment for 24 hours together with or without OA (100 nM) for 4 hours before harvesting (*t*-test; n = 3, p>0.05). F. Cell lysates from (E) were immunoblotted with anti-cleaved caspase 3 or YAP2 antibody.

The identification of a signaling link between PP1 and YAP2 that leads to the nuclear accumulation of YAP2 raised the possibility that PP1-induced YAP2 dephosphorylation might modulate YAP2-dependent gene transcription and cell survival. We took advantage of 3*Sd-LUC reporter system [23] to examine the effect of PP1 expression on the YAP2 co-transcriptional activity. While PP1A expression significantly increased YAP2 activity, OA treatment led to the inhibition of PP1A-induced upregulation of YAP2's transcriptional activity (Figure 4A). *CTGF* (connective tissue growth factor) is one of the putative YAP2-TEAD target genes [35], [36], [37]. PP1A expression further increased YAP2-mediated upregulation of *CTGF* expression in quantative-RT-PCR assays (Figure 4B). Taken together, PP1A increases the YAP2-mediated transcriptional activation.

It has been demonstrated that YAP functions as an oncoprotein in a panel of cancers including ovarian, breast and prostate cancer [17], [18], [19], [20]. In according with those findings, expression of YAP2 protected A2780 cells from cisplatin-induced cell death (Figure 4C). Similar to S127A mutant YAP2, co-expression of PP1A further increased YAP2-mediated cell survival (Figure 4C), suggesting PP1A act synergistically with YAP2 to enhance ovarian cancer cell survival and dephosphorylation of YAP2 increased its pro-survival capability. Accordingly, while stably expressed YAP2 in A2780 cells inhibited cisplatin-induced Caspase-3 cleavage, OA treatment abolished YAP2's protective effect (Figure 4D). In contrast to YAP2 expression, YAP2 knockdown by using shRNA increased DNA damage-induced cell death (Figure 4E). OA treatment failed to increase cisplatin-induced cell death as well as Caspase-3 cleavage (Figures 4E and 4F). Collectively, these data suggest that YAP2 dephosphorylation by PP1A enhances YAP2 transcriptional activation, and PP1A acts synergistically with YAP2 to promote cell survival in ovarian cancer cells.

In this study, we have discovered a novel regulatory mechanism of YAP2 by PP1-meidated dephosphorylation. The identification of PP1A as the YAP2 complex protein suggests PP1A plays an important role in the regulation of the biological function of YAP2 including pro-survival capability.

Our study implicates that the phosphorylation levels of YAP2 is dynamically regulated in the process of cell death. Protein phosphatase PP1 reduces YAP2 phosphorylation at serine 127 and disrupts the association with 14-3-3 proteins, which results in the nuclear accumulation and transcriptional activation of YAP2. Activation of YAP2-dependent transcription protects ovarian cancer cells from cisplatin-induced cell death. Since multiple lines of evidences have demonstrated that Hippo/MST-Yki/YAP2 signaling is conserved in mammals and *Drosophila* [1], an important goal of future studies is to determine whether Yki is regulated by PP1 in *Drosophila*.

In agreement with the findings that Yap1 and TAZ are dephosphorylated and activated by PP2 and PP1, respectively [39], [40], we elucidate that PP1 dephosphorylates YAP2 at serine 127. However, it has been reported that there are multiple phosphorylation sites on YAP2 proteins induced by Lats or CK (Creatine kinase) kinase [41]. CK phosphorylates YAP2 and induces its degradation *via* TrCP [41]. Our findings also raise the possibility that PP1 might be involved in the removal of other phosphorylation sites, namely the CK-mediated phosphorylation, and inhibit the protein degradation of YAP2. In support of this hypothesis, we found that PP1A expression stabilized YAP2 protein in our CHX (cyclohexamine)-chase experiments (Figures 3F and 3G).

Tight junction related proteins, such as AMOLTL1/2 and Patj, have been shown to modulate YAP2 function in the process of cellular proliferation and homeostasis [35], [36]. Given the fact that PP1A and YAP2 forms a physical complex together with other appreciated YAP2 interacting proteins including tight junction related proteins, it will be interesting to investigate how protein phosphatase PP1 regulates YAP2 function synergistically with these tight junction proteins in the process of cellular polarity establishment and homeostasis maintenance.

## Materials and Methods

### Reagents and Cell Culture

HeLa cells and Ovarian cancer cells (ATCC) were cultured at 37°C and 5% CO_2_ Dulbecco's modified Eagle's medium (Invitrogen) supplemented with 10% fetal bovine serum (Invitrogen), 100 U/ml penicillin and streptomycin (Gibco). Antibodies of FLAG (Sigma), Myc (Santa Cruz), PP1A (Epitomics), GST (Santa Cruz), YAP2 (Santa Cruz), Phospho-Ser127-YAP2 (Cell Signaling), Erk1/2 (Cell Signaling), Actin (SIGMA), GAPDH (SIGMA) were purchased. Cisplatin, okadaic acid and CHX (Cyclohexamine) were purchased from Sigma.

### Expression Constructs

3xFLAG- tagged MST1 construct was subcloned in the pCMV10 expression vector. Myc-tagged YAP2 and Myc-tagged Lats2 constructs were subcloned into the pCMV-Myc expression vector. GFP-YAP2 construct is in the GFP-C2 expression vector and GST-YAP2 and GST-PP1A construct is in the pGEX expression vector. Ad-YAP2, Ad-GFP, Ad-YAP2 shRNA or Ad-U6 adenovirus was generated to infect the cells. YAP2 shRNA targeting sequence: GACAUCUUCUGGUCAGAGA [13], the sequence was cloned into Ad Basic vector under U6 promoter.

### Tandem-affinity purification (TAP) of YAP2 Protein Complex

TAP assay has been previously described [42], [43]. Briefly, 4 liters of suspension HeLa cells that stably transfected with pOZ-FLAG-HA-YAP2 were harvested in 50 ml hypotonic buffer (1M Tris-HCl pH 7.3, 3M KCl, 1M MgCl_2_) containing protease and phosphatase inhibitors. Cells were homogenized and the nuclear or cytoplasmic fractions were collected respectively. YAP2 was immunoprecipitated from the nuclear or cytoplasmic extracts by using the anti- FLAG and HA beads sequentially and the final eluate was digested with trypsin and analyzed by mass spectrometry.

#### Density gradient centrifugation

The precipitated proteins from TAP were centrifuged in 10%–40% gels as described [44].

### Immunoprecipitation and Immunoblotting

Immunoprecipitation and immunoblotting analysis was carried out as described previously. Briefly, cell lysates were incubated with the indicated antibodies in the presence of 15 µL of protein A-protein G (2∶1)-agarose beads for 2 hours at 4°C. After washed four times, the immunoprecipitates were subjected to electrophoresis. Protein expression was examined by probing Western blots of total cell lysates or immunoprecipitates with the appropriate antibodies as noted in the figure legends. Detection of band intensity was carried out with the ECL Western blotting Analysis System.

### In vitro Kinase Assay and dephosphorylation assay

*In vitro* kinase reactions were carried out by incubating immunprecipitated Lats and the recombinant GST-YAP2 for 30 min at 30°C in the presence of 3 µM cold ATP in 30 µl of buffer containing 20 mM HEPES (pH 7.4), 10 mM MgCl_2_, 10 mM MnCl_2_, 1 mM DTT [45]. *In vitro* dephosphorylation assay was performed by incubating the kinase reaction mixture with the recombinant GST-PP1 or GST only protein for 1 hour at 37°C in buffer containing 50 mM HEPES, 100 mM NaCl, 1 mM MnCl_2_, 2 mM DTT, 0.1 mM EGTA and 0.025% Tween 20 followed by the SDS-PAGE gel and immunoblotting with the indicated antibodies.

### Luciferase Reporter Assay

Luciferase activity was measured as described [23] according to the manufacturer's guidelines. HeLa cells were cultured in 24-well plates. 3*Sd binding site artificial-luciferase reporter was co-transfected with the indicated expression plasmids. 36 hours after transfection, cells were harvested, and luciferase activity was measured. All luciferase activities were normalized to Renilla and repeated for three times.

#### RNA isolation and real-time PCR assay

Total RNA was isolated from cultured cells using Trizol reagent (Invitrogen). cDNA was synthesized by reverse transcription using oligo dT as the primer and proceeded to real-time PCR with gene-specific primers in the presence of SYBR Premix Ex Taq (DRR041A; TaKaRa). The relative abundance of mRNA was calculated by normalization to glyceraldehyde-3-phosphate dehydrogenase (GAPDH) mRNA. Primers sequence used for CTGF is 5′-gcttaccgactggaagacacg and 3′- cggatgcactttttgccctt.

### Apoptosis Analysis

Cell death assays were performed according to the manufacturer's guidelines (BD Pharmingen). Briefly, cells were cultured in 6-well plate and grown in DMEM medium supplemented with 10% fetal bovine serum for 24 hours, then treated with cisplatin (20 µM) for 24 hours, both floated and attached cells were collected. Cells were washed with PBS, and resuspended in binding buffer containing 5 µL Annexin V followed by flow cytometry (Beckman Coulter). All the experiments were performed three times.

### Statistical analysis

Statistical analysis of the data was performed with a two-tailed Student's *t*-test. Data are presented as the mean ± SEM except for analyses of luciferase assays where mean ± SD is shown. *P <0.05 or **P<0.01 denotes statistical significance.
